# Affordable Rental Housing and Older Adults: A Case Study for Community Development Initiatives

**DOI:** 10.1093/geroni/igaa057.2491

**Published:** 2020-12-16

**Authors:** Muhammad Qureshi, Atiya Mahmood, Ghazaleh Akbarnejad, Rahil Adeli, Dana Sharon

**Affiliations:** 1 Simon Fraser University, Vancouver, British Columbia, Canada; 2 Brightside Community Homes Foundation, Vancouver, British Columbia, Canada

## Abstract

Older adults living in rental housing with limited income are at-risk for experiencing life-course disruptions, including social isolation, loneliness and homelessness. To address these needs, one Vancouver-based affordable housing provider for older adults has implemented community development initiatives (CDIs), including resident-led community gardens, workshops, and social events. Based on data from three focus groups, resulting in a total of fifteen participants, this study explored the efficacy of five different CDIs in supporting residents’ social connection and sense of community. Findings revealed that CDIs contribute to increased social engagement and inclusion of older adults living in affordable rental housing. In particular, resident-led community gardens were identified as the most impactful CDI in supporting both social engagement and inclusion, with the added benefit of addressing resident’s food insecurity. We discuss how CDIs can be implemented in various housing settings for low-income older adults as an effective method of supporting aging-in-the-right place. Part of a symposium sponsored by the Environmental Gerontology Interest Group.

